# Impact of Foodborne Disease in Taiwan during the COVID-19 Pandemic

**DOI:** 10.3390/medicina60030505

**Published:** 2024-03-19

**Authors:** Ya-Lu Yang, Cheng-Chueh Chen, Pi-Wei Chin, Chun-Gu Cheng, Chun-An Cheng

**Affiliations:** 1 Department of Emergency Medicine, Tri-Service General Hospital, Songshan Branch, Taipei 10581, Taiwan; 2 Department of Health Promotion and Health Education, National Taiwan Normal University, Taipei 10610, Taiwan; 3 Department of General Surgery, China Medical University Beigang Hospital, Yunlin 65152, Taiwan; 4 Department of Nursing, Ministry of Health and Welfare, Hualien Hospital, Hualien 97061, Taiwan; 5 Department of Emergency Medicine, Taoyuan Armed Forces General Hospital, Taoyuan 32549, Taiwan; 6 Department of Emergency Medicine, Tri-Service General Hospital, National Defense Medical Center, Taipei 11490, Taiwan; 7 Department of Neurology, Tri-Service General Hospital, National Defense Medical Center, Taipei 11490, Taiwan

**Keywords:** foodborne diseases, COVID-19 pandemic, surveillance

## Abstract

*Background and Objectives*: The coronavirus disease 2019 (COVID-19) pandemic originated in Wuhan, China, in December 2019, the first case diagnosed since January 2020 in Taiwan. The study about the potential impact of the COVID-19 pandemic on event, location, food source, and pathogens of foodborne disease (FBD) is limited in Taiwan. Our aim in this study is to investigate FBD in the context of the COVID-19 pandemic. *Materials and Methods*: We collected publicly available annual summary data from the FBD dataset in the Taiwan Food and Drug Administration and Certifiable Disease on reported FBD in Taiwan from 2019 to 2020. We used logistic regression to evaluate changes in the occurrence or likelihood of FBD cases and Poisson regression to examine the relative risk (RR) between FBD and climate factors. *Results*: Similar events occurred in 2019 and 2020, but the total number of FBD cases decreased from 6935 in 2019 to 4920 in 2020. The places where FBD decreased were in schools, hospitals, outdoors, vendors, and exteriors. The top place in FBD shifted from schools to restaurants. The top food source for FBD has changed from boxed food to compound food. *Bacillus cereus* and *Salmonella* emerged as the top two observed bacterial pathogens causing FBD. The risk of FBD cases increased with a higher air temperature, with an RR of 1.055 (1.05–1.061, *p* < 0.001) every 1 °C. *Conclusion*: The incidence of FBD decreased significantly during the COVID-19 pandemic in Taiwan. This decline may be attributed to protective measures implemented to control the spread of the virus. This shift in locations could be influenced by changes in public behavior, regulations, or other external factors. The study emphasizes the importance of understanding the sources and effectiveness of severe infection prevention policies. The government can use these findings to formulate evidence-based policies aimed at reducing FBD cases and promoting public health. Consumers can reduce the risk of FBD by following safe food handling and preparation recommendations.

## 1. Introduction

A cluster of pneumonia cases of unknown origin was discovered in Wuhan, Hubei Province, China, in December 2019, and a novel coronavirus infection was officially diagnosed on 7 January 2020, later named SARS-CoV-2 (COVID-19). The clinical manifestations of most cases were fever and muscle aches, and some severe patients had difficulty breathing. Chest radiography showed infiltrative lesions in both lungs combined with pneumonia [1]. The epidemic quickly spread around the world, and human-to-human transmission was subsequently confirmed [2,3]. The World Health Organization (WHO) declared the epidemic to constitute a Public Health Emergency of International Concern (PHEIC) on 30 January 2020 [4]. Taiwan detected its first case on 21 January 2020, and has implemented and maintained strict intervention measures, such as large-scale epidemiological surveys, rapid diagnosis, wearing masks, washing hands frequently, safe social distance, and timely clinical treatment of COVID-19 patients in Taiwan. Triage and provide appropriate medical treatment. Promptly provide corresponding medical treatment and hospitalization for serious cases with a high mortality rate [5,6].

Foodborne disease (FBD) is recognized as a critical public health concern. The health impacts and economic costs of unsafe food, as well as the impact on global health, trade, and development, are considered to be enormous. Hundreds of millions of cases of FBD occur worldwide every year, costing billions of dollars in losses and contributing to the deterioration of the health quality of the world’s population [7,8]. FBD can occur at any stage of the food and beverage production, transportation, and consumption chain. It is caused by food contamination, which can result from various forms of environmental contamination, including water, soil, or air contamination, as well as unsafe food storage and processing. FBD encompasses illnesses caused by parasites, chemicals, and pathogens that can contaminate food at different points in the food production and preparation process. The WHO defines FBD as an infectious disease resulting from the ingestion of food containing pathogenic microorganisms or their toxins. Infection occurs through a variety of other means (e.g., parasites, autoimmunity, toxins, heavy metals, stress, etc.) [9]. Common symptoms of FBD include nausea, vomiting, fever, abdominal pain, diarrhea, a lack of appetite, symptoms of dehydration, and neurological symptoms [10]. The range and severity of symptoms may vary depending on the contaminant. The surveillance system for FBD identified higher incident places, high-risk foods, and FBD of specific pathogens. The government surveyed the data to identify vulnerable groups and promote food safety programs by monitoring FBD trends and identifying and controlling outbreaks to reduce the burden. Half of FBD cases with identifiable leading pathogens were non-typhoid *Salmonella*, norovirus, and *Vibrio parahaemolyticus* during 2012–2014 in Taiwan [11]. The top two source locations of FBD were schools and restaurants. The top two food source classifications of FBD were boxed meals and compounded foods. The top two bacterial classifications of the observed FBD were *Bacillus cereus* and *Staphylococcus aureus* during 2014–2018 in Taiwan [12].

The residents worried about the infection, panicked, and bought masks, alcohol, and food for storage. The mandatory mask wearing and hand washing seemed to be the influences on the decrease in FBD. The overbuying of food must be stored in the refrigerator or room air of the home, but inadequate storage methods or overdue orders could increase FBD incidents of the compound food and cake or candy; raw and cooked food contaminated would increase some type of FBD. Lockdown measures such as school closures, stay-at-home orders, working at home, and protective measures (including social distancing, wearing masks, and increasing hand hygiene) have changed lifestyles and behavioral and social habits [13]. The effect of FBD in the COVID-19 era in Taiwan must be surveyed.

The aim of this study was to evaluate the change in FBD in Taiwan under the COVID-19 epidemic compared with the previous situation. Based on changes in FBD locations and food sources, the possible mechanisms of changes in diet and consumption behavior were explored. Preventive measures against viruses and their potential consequences for food safety. It needs further access to the association of mechanisms in the future.

## 2. Materials and Methods

**Figure 1:**
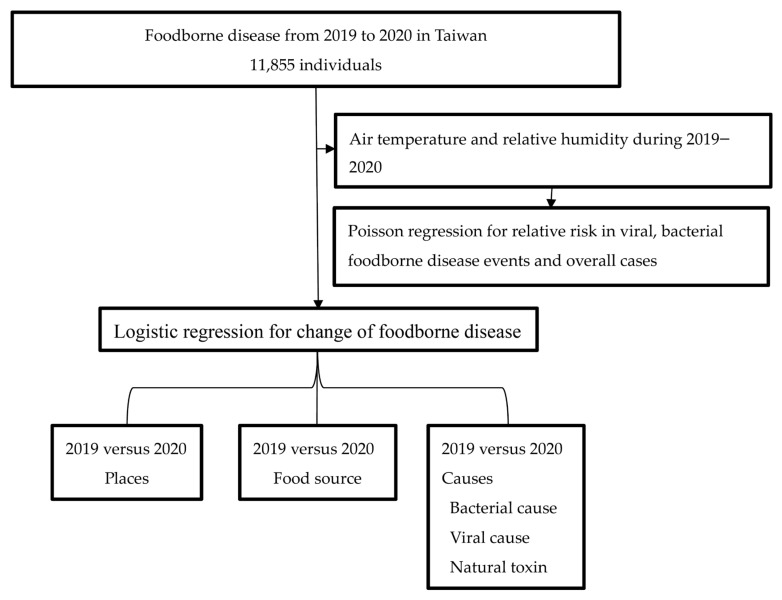
The flowchart of this study.

When more than two patients suffer from diarrhea, vomiting, etc. symptoms while eating the same food, medical institutions must report suspected food poisoning to the health bureaus of the local government. They must investigate and take relevant prevention and control measures. It could apply to the Department of Disease Control for an epidemiological investigation to clarify the route of infection and the causative substances and foods that may cause food poisoning. Utilizing the annual FBD dataset from the Taiwan Food and Drug Administration and Certifiable Disease for the years 2019 and 2020 allows for a comprehensive analysis. The criterion for defining FBD as more than two patients suffering from symptoms related to the same food is a suitable approach for identifying potential outbreaks. The proportion of places and food sources and the incidence of FBD causes were calculated [14]. There were 236 million residents in 2019 and 235.7 million residents in 2020 in Taiwan. The incidence of FBD causes was calculated by FBD cases divided by the overall number of residents. The change in places, sources, and causes of FBD in 2020 compared with the data in 2019 was performed by logistic regression. The bacterial FBD events, viral FBD events, and overall FBD cases were collected from the Taiwan Food and Drug Administration every month from 2019 to 2020. The mean air temperature and relative humidity data were obtained and sourced from the Taipei Weather Administration every month from 2019 to 2020 [15]. The log-linear model was performed for the relationship between FBD and climate factors. The flowchart of this study is shown in Figure 1. This study was approved by the Institutional Review Board of the Tri-Service General Hospital of TSGHIRB-C202305039.

The proportion of places, media, foods, and causes was evaluated by the Chi-square test. The logistic regression was utilized to calculate odds ratios (ORs), providing insights into the association between FBD and specific places, food sources, and causes. The Poisson regression was used to identify the relative risk (RR) of air temperature and relative humidity in viral, bacterial FBD events, and overall FBD cases. The significant statistical difference was defined as *p* < 0.05. All statistical analyses were performed using SPSS version 21.

## 3. Results

There were similar FBD events between 2019 (*n* = 502) and 2020 (*n* = 506) (*p* = 0.072) (Supplementary Table S1. The events of place in foodborne disease during 2019-2020 in Taiwan). The events of place in foodborne disease during 2019–2020 in Taiwan.); however, the FBD cases decreased by 29.06%, from 6935 cases in 2019 to 4920 cases in 2020. The top three primary incident bacterial classifications of the observed FBD were *Bacillus cereus*, *Salmonella,* and *Staphylococcus aureus*. The top two primary natural toxin classifications of the FBD were plants and histamines. The incidence of FBD in military facilities, fruits and vegetables, and *Staphylococcus aureus* was increased in our study.

The number of FBD cases decreased in schools, hospitals, outdoors, venders, and exteriors. The FBD incident decreased in schools with odds ratio (OR) 0.42 (95% confidence interval [C.I.]: 0.39–0.45, *p* < 0.001), hospitals with OR 0.06 (0.03–0.13, *p* < 0.001), outdoor with OR 0.1 (0.04 to 0.23, *p* < 0.001), venders with OR 0.28 (95% C.I.: 0.2–0.38, *p* < 0.001) and exterior with OR 0.43 (95% C.I.: 0.28–0.65, *p* < 0.001). There were 2197 FBD cases from 1593 cases in restaurants, 114 FBD cases from 19 cases in military facilities, and 124 FBD cases from 62 cases in prisons. The proportion of FBD cases in offices increased to 7% (343), up from 5.9% (407). The cases increased in restaurants with OR 2.71 (95% C.I.: 2.5–2.93, *p* < 0.001); offices with OR 1.2 (95% C.I.: 1.04–1.39, *p* = 0.015), military facilities with OR 8.63 (95% C.I.: 5.31–14.05, *p* < 0.001); and prisons with OR 2.87 (95% C.I.: 2.11–3.9, *p* < 0.001). (Table 1).

**Figure 2:**
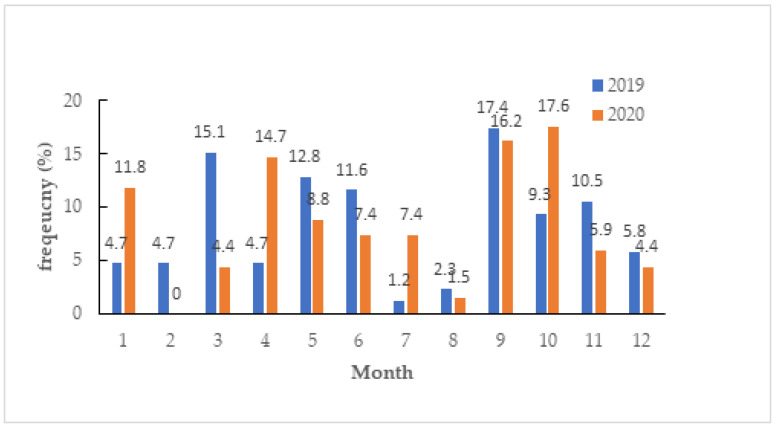
The events of foodborne disease in school during 2019 (*n* = 86) and 2020 (*n* = 68) (*p* = 0.022).

The change in FBD events in school is shown in Figure 2. The top two FBD events were 15.1% in March and 17.4% in September during 2019. The top two FBD events were 16.2% in September and 17.6% in October during 2020. It decreased from 86 to 68 FBD events by 20.93%.

**Figure 3:**
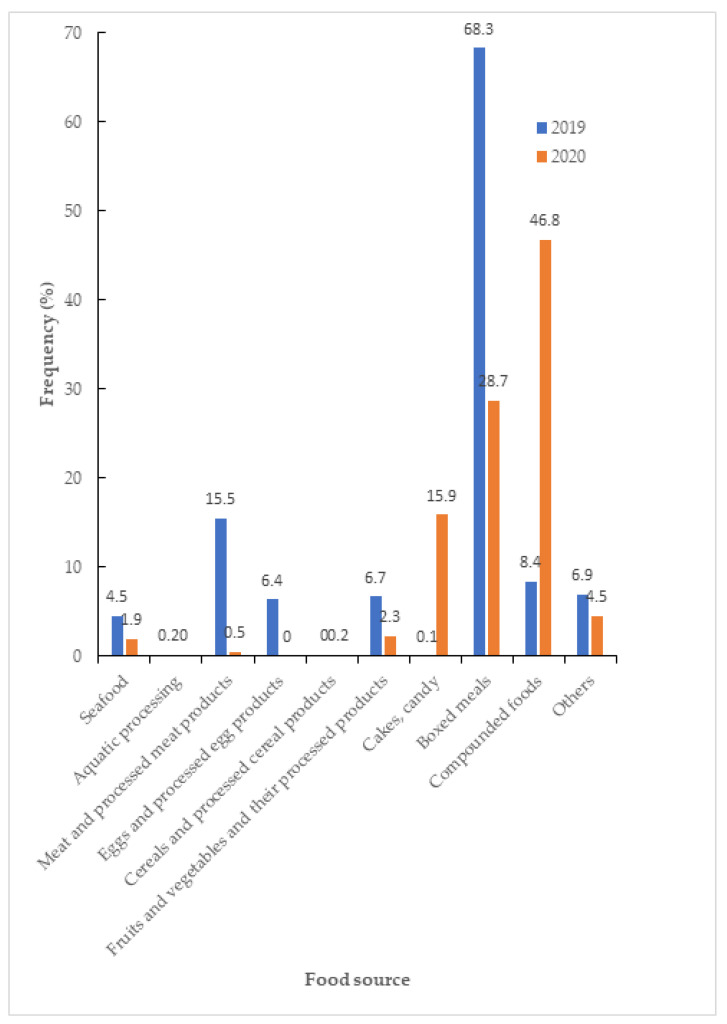
The food source of foodborne diseases from 2019 to 2020 in Taiwan (*p* < 0.001).

**Figure 4:**
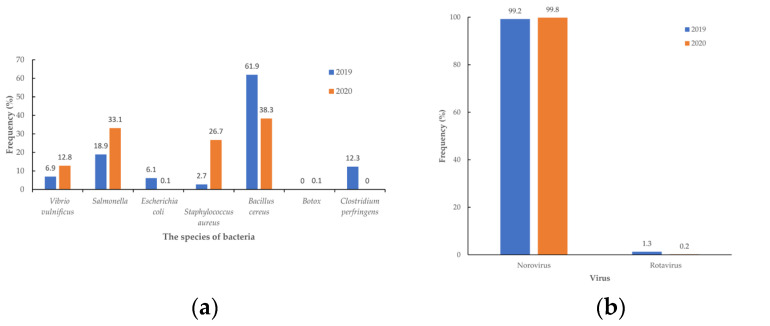

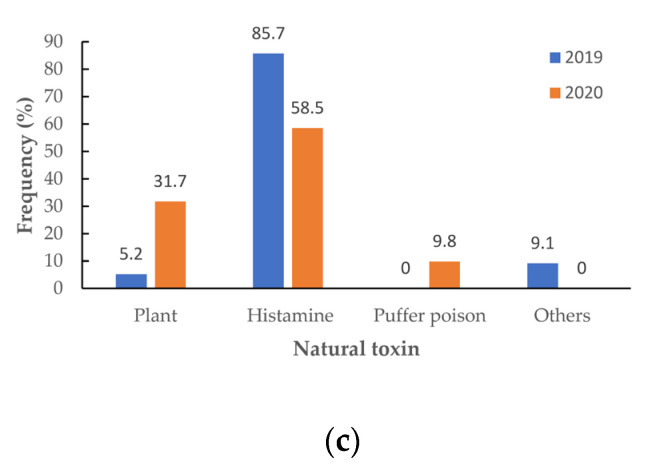
The bacterial cause (**a**), viral cause (**b**), and natural toxin (**c**) of foodborne disease from 2019 to 2020 in Taiwan.

The food source for FBD events was similar (Supplementary Table S2. The food source of foodborne diseases from 2019 to 2020 in Taiwan). The food source of FBD cases was significantly reduced in boxed meals and processed products but increased in cakes, candy, and compounded foods (Figure 3). The change in events of bacterial pathogens was increased in *Vibrio vulnificus* and *Salmonella* but decreased in *Escherichia coli*, *Staphylococcus aureus,* and *Bacillus cereus* (*p* = 0.032) (Supplementary Table S3. The events about causes of foodborne diseases from 2019 to 2020 in Taiwan.). The incidence of bacterial FBD decreased from 14.2/100,000 to 6.21/100,000 (*p* < 0.001) and the incidence of virus FBD decreased from 9.44/100,000 to 6.88/100,000 (*p* < 0.001). The incidence of *Bacillus Cereus* decreased from 8.79/100,000 to 2.38/100,000 (*p* < 0.001), the incidence of *S. aureus* decreased from 3.82/100,000 to 1.66/100,000 (*p* < 0.001), the incidence of *Salmonella* decreased from 2.68/100,000 to 2.05/100,000 (*p* < 0.001), the incidence of *Vibrio vulnificus* decreased from 0.98/100,000 to 0.13/100,000 (*p* < 0.001) and the incidence of *Escherichia coli* decreased from 0.86/100,000 to 0.01/100,000 (*p* < 0.001). The percentage of bacterial FBD cases was reduced in *Bacillus cereus* and *Escherichia coli* but increased in *Vibrio vulnificus*. *Salmonella* and *Staphylococcus aureus* (Figure 4a). The percentage of viral FBD cases was reduced in rotavirus (Figure 4b). The percentage of natural toxin FBD cases was reduced in histamine but increased in plants and puffer poison (Figure 4c). The incident bacterial classifications of the observed FBD were decreased risk in *Bacillus cereus* (OR 0.38 [95% C.I.: 0.34 to 0.43], *p* < 0.001]) and *Escherichia coli* (OR 0.02 [95% C.I.: 0.01–0.09], *p* < 0.001]). But increased risk in *Vibrio vulnificus* with OR 1.98 (95% C.I.: 1.62 to 2.43, *p* < 0.001) and *Salmonella* with OR 2.13 (95% C.I.: 1.85 to 2.44, *p* < 0.001). (Table 2).

The incidence of norovirus decreased from 9.37/100,000 to 6.86/100,000 (*p* < 0.001), and the incidence of rotavirus decreased from 0.12/100,000 to 0.02/100,000 (*p* < 0.001). The incidence of natural toxin decreased from 0.33 to 0.17/100,000; the incidence of plants increased from 0.02 to 0.06/100,000; and the incidence of histamine decreased from 0.28 to 0.1/100,000. The incident viral classifications of the observed FBD were decreased in rotavirus (OR 0.19 [95% C.I.: 0.07–0.55, *p* = 0.002) (Table 2). The residents wear a mask and wash their hands frequently, which reduces the bacterial and viral FBD.

**Figure 5:**
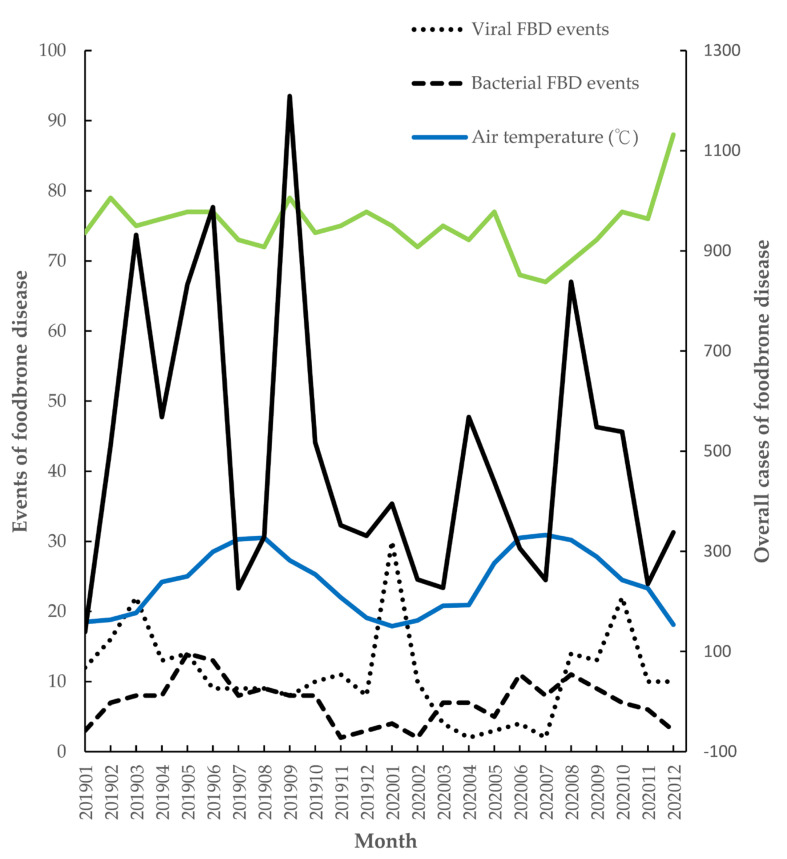
The events of viral and bacterial foodborne disease, the case of foodborne disease, and climate in Taiwan.

There were 11.04 ± 6.64 viral FBD events, 7.13 ± 3.25 bacterial FBD events, and 494 ± 280 FBD cases during 2019 to 2020 in Taiwan. The mean air temperature was 24.16 ± 4.62 °C, and the mean relative humidity was 74.96 ± 4.13% in the two years in Taipei. The top viral FBD events were 22 events in March during 2019 and 30 events in January during 2020. The spikes in mean air temperature were in August 2019 and July 2020; the nadirs of mean air temperature were in January 2019, January 2020, and December 2020. The spike in bacterial FBD events was in May 2019, June 2020, and August 2020. The spike in viral FBD events was in March 2019, January 2020, and October 2020. The higher cases of overall FBD were in March, June, September, 2019 and April, August, 2020 (Figure 5). The risk of viral FBD events increased with a lower air temperature with relative risks (RR) 0.954(0.926–0.984, *p* = 0.003) every 1 °C arise and the risk of bacterial FBD events increased with a higher air temperature with RR 1.078(1.034–1.124, *p* < 0.001) every 1 °C. The risk of FBD cases increased with a higher air temperature with RR 1.055(1.05–1.061, *p* < 0.001) every 1°C (Table 3).

## 4. Discussion

Our study confirms a notable decrease in the incidence of FBD during the COVID-19 outbreak in Taiwan. There was a significantly decreased incidence of FBD in schools, hospitals, outdoors, vendors, and exteriors. The findings emphasize the importance of preventive measures such as mask wearing, frequency of hand washing, and public health policies to maintain social distance in reducing the incidence of COVID-19 infections in the COVID-19 era. They also significantly reduced the FBD incidents. It is worthy of attention for the healthcare staff to understand the effect of the prevention policy on severe respiratory infections for the benefit of food safety.

Outbreaks of FBD can impact productivity, with trade putting pressure on healthcare systems. The incidence of infections transmitted through food decreased by 26% during 2020 in the United States [16]. Our study found 29.06% of cases decreased in Taiwan. The cases of FBD were 6935, 4920, 5823, 4495 from 2019 to 2022; there was a 35.18% decrease in the most severe COVID-19 outbreak during 2022 [14]. Widespread public health interventions were implemented, and inside use was restricted in restaurants during the COVID-19 pandemic [5]. Implementations to prevent SARS-CoV-2 transmission might have contributed to this decrease.

It is estimated that in 9.4 million cases, 1 in 6 Americans suffers from FBD, resulting in 128,000 hospitalizations and 3000 deaths, with the majority of patients being children under five years of age or school-age children in the United States [17]. According to recent estimates from the WHO, approximately 600 million cases of FBD and 420,000 related deaths were caused by 31 FBD hazards each year in 2017 [9,18]. According to data from theWHO, the most common causes of cases of FBD diarrhea are Norovirus and *Campylobacter* spp., but most cases resulting in death are due to *Salmonella* spp. [18]. In the past few years, FBD in military facilities, infections with fruits, vegetables, and processed foods, and *Bacillus cereus* infections have been on the rise year by year, while *Vibrio vulnificus* and *Salmonella* infections have been on the decline [12]. 

The nutritious lunches were provided in elementary schools and junior and senior high school, and the students received lunch boxes transferring by food takeaway delivery platform or self-prepared in senior high school. According to the class suspension criteria for COVID-19 in 2020, COVID-19 students, teachers, and school staff need to be quarantined for 14 days, and if two COVID-19 patients are in school in 14 days, then the school must be closed. In order to reduce the risk of cluster infections and safeguard the health and learning rights of teachers and students in schools, There was a 25% decrease in FBD events in schools in 2020. The percentage of schools in FBD places was 57.80%, 36.30%, 48.90%, and 35.60% from 2019 to 2022 (*p* < 0.001) [14]. The period during which schools and public and private kindergartens at all levels across the country are suspended from 19 May 2021 extended to 27 July 2021. The government of Taiwan has stopped face-to-face classes in schools, and students have switched to online learning at home. All students study remotely at home and cannot attend school. Video instruction, less eating outside, and contact together may lead to decreased FBD rates in schools under the COVID-19 pandemic. It showed a persistent decrease in school attendance during the COVID-19 pandemic.

There was a significant decrease in hospital visits in Taiwan during the COVID-19 era [19,20]. In the early days of the epidemic, the number of daily emergency department visits was significantly reduced, and the quality of medical care was greatly improved [20]. The percentage of hospitals in FBD places was 20.90%, 0.10%, 0.60%, and 1.40% from 2019 to 2022 (*p* < 0.001) [14]. It showed a persistent decrease in vendors during the COVID-19 pandemic. Lifestyles have changed due to maintaining a socially safe distance of 1.5 m, staying-at-home orders, and limiting dining out to avoid person-to-person contact. Less contact may lead to decreased FBD rates in hospitals during the COVID-19 pandemic. The percentage of venders in FBD place was 3.20%, 0.90%, 0.70%, and 0.80% from 2019 to 2022 (*p* < 0.001) [14]. It showed a persistent decrease in vendors during the COVID-19 pandemic. The citizens cook food frequently for themselves and eat less outside. 

Significantly increased incidents of FBD were noted in restaurants, offices, prisons, and military facilities during the COVID-19 pandemic. The percentage of military facilities in FBD places was 0.30%, 2.30%, 1.80%, and 5.90% from 2019 to 2022 (*p* < 0.001) [14]. It showed a persistent increase in military facilities during the COVID-19 pandemic. The people ate together in restaurants, offices, military facilities, and prisons, which increased the incidents of FBD. The food poisoning process may involve cluster infections caused by eating the same contaminated food at the same location [19]. Although the cooking staff obeyed hygienic habits by wearing masks, gloves and washing hands with alcohol, they cleaned and disinfected the environment after the COVID-19 outbreak. Most of the FBD disease burden comes from microbial hazards, due to a large increase in the consumption of risky foods (livestock, fish products, and agricultural products) [7]. But restaurants, military facilities, and prisons buy larger amounts of food once for preparing several days of diets, which are stored in their places for days if cross-contamination of raw and cooked food, insufficient temperature in the refrigeration (frozen) room, and eating meals prepared by sick staff may cause pathogenic microorganisms to breed in the food. Storage at room temperature for too long or leftovers in offices may cause FBD in offices.

There was a higher incidence (15.9%) of cakes or candy from 2019 to 2020. the residents bought a larger amount of food for the COVID-19 outbreak due to improper preservation or overdue food. The increased risk of incidents of the compounded foods of boxed meals or sandwiches in convenience stores (46.8%) from 2019 to 2020. The percentage of compounded foods in food sources was 8.40%, 46.80%, 8.50%, and 40.90% from 2019 to 2022 (*p* < 0.001) [14]. It increased in 2020 and 2022 compared with 2019. These foods have diverse ingredients, they may be contaminated during food transfer and storage. The higher proportion of persons who ate outside of Taiwan; meal boxes were convenient meals for persons in Taiwan; left-over or contaminated foods can directly lead to food poisoning [12]. The percentage of boxed meals in food sources was 68.30%, 28.70%, 64.50%, and 46.40% from 2019 to 2022 (*p* < 0.001) [14]. It decreased in 2020 and 2022 compared with 2019. The previous Korea study found bacterial growth that causes harm when temperatures rise. After 90 min, the temperature of the frozen meat reaches the danger zone (5–60 °C), which is associated with bacterial growth [21].

The number of bacterial-caused FBD decreased, as did the risk of *Escherichia coli* and *Bacillus cereus*. The cooking staffs paid more attention to diet hygiene caused number and risk reduction. The percentage of *Bacillus cereus* in bacteria showed a persistent decrease of 61.90%, 38.30%, 56.80%, and 47.40% from 2019 to 2022 (*p* < 0.001) [14]. The percentage of *Escherichia coli* decreased to 1% during 2020 and 2021 but increased to 19.1% in 2022 (*p* < 0.001). Maintaining the stability of *Vibrio vulnificus* and *Salmonella* FBD has been effective after close hygiene monitoring, but there is increased risk after the COVID-19 epidemic situation. For those who cook at home frequently or order seafood, eggs, and processed egg products, the risk of these two bacterial FBDs arises from improper cooking practices with foods that are not really cooked or contamination with raw foods. The percentage of *Vibrio vulnificus* in bacteria showed a persistent increase of 6.90%, 12.80%, 9.70%, and 15.80% from 2019 to 2022 (*p* < 0.001) [14]. The percentage of *Salmonella* in bacteria showed 18.80%, 33.00%, 21.32%, and 13.10% from 2019 to 2022 (*p* < 0.001) [14], increased to 2021, but decreased in 2022.

The number of viral-caused FBDs decreased. It showed decreased risk in rotavirus with OR 0.19 but no change in norovirus. This suggests a lower likelihood of contracting rotavirus-related illnesses. The residents wear masks compulsorily, and some individuals wear gloves in their hands, causing virus infections to decrease.

The nature toxin cause of FBD showed the plants increased with OR 8.5, which suggests a substantially higher risk of FBD associated with nature toxins in 2020. Histamine decreased by 0.24 in 2020 compared with 2019, which implies a lower risk of FBD associated with histamine in 2020. Because of the epidemic situation, more individuals cooked vegetables at home during this period. Additionally, some people occasionally consume or accidentally pick poisonous plants, which may have contributed to the increase in FBD cases associated with nature toxins.

The mean air temperature changed from 24.1 °C to 24 °C in Taipei. The risk of viral FBD events increased while a lower air temperature with an RR of 0.9 with every 1 °C arose. The risk of bacterial FBD events increased with higher air temperatures with an RR of 1.07 with every 1 °C. The risk of other FBD cases increased with a higher air temperatures, with an RR of 1.03 with every 1 °C. The events of pathologic viruses in FBD are associated with low temperatures; the events of pathologic bacteria in FBD; and overall FBD cases are associated with high temperatures. The mean values of relative humidity were higher than 60% due to island weather in Taiwan, and the Poisson regression showed no significant statistical difference.

In Italy, 5.6% of FBD surveys were analyzed, and more than half of the positive samples involved meat [22]. Microbial contamination of food can occur at any stage of the food chain, from farm to fork. *Staphylococcus aureus* causes FBD due to contamination in unhygienic situations. Therefore, good hygiene and production practices must be followed throughout the food chain to prevent food contamination caused by microorganisms, leading to high morbidity and mortality among consumers [23,24]. The enterotoxin secreted by *Staphylococcus aureus* has heat-resistant properties and is not easily removed after cooking. FBD of *Staphylococcus aureus* caused approximately 241,000 cases of illness annually in the United States in 2011.Symptoms include nausea, vomiting, and abdominal cramping with or without diarrhea. Preventive measures include safe food handling and processing practices, maintaining cold chains, adequate cleaning and disinfection of equipment, preventing cross-contamination in homes and kitchens, and preventing farm-to-table contamination [25]. The percentage of *Staphylococcus aureus* in bacteria was 26.90%, 26.70%, 37.80%, and 60.10% from 2019 to 2022 (*p* < 0.001) [14], it increased from 2021. Some bacteria (shigellosis and listeriosis), parasites (amebiasis and toxoplasmosis), and viruses (hepatitis A and E) were classified as notifiable infectious diseases by the Taiwanese Centers of Disease Control [26]. Hepatitis A (cases from 107 to 74) and amoebic dysentery (cases from 352 to 250) have declined during COVID-19 beginning, with preventive measures and border controls causing the decline.

Histamine FBDs account for half of the natural toxin and often occur in inappropriate environments such as 15–20 °C, which can lead to the proliferation of bacteria on the surface or in the intestines, converting the histine acid in the fish into histamine. Although cooking can kill bacteria, it cannot eliminate the histamine produced. After eating foods containing histamine, the human body may experience symptoms such as headaches, hypotension, urticaria, vomiting, and diarrhea. It was reduced in histamine during the COVID-19 era. Poor storage conditions and germination induced plant FBD incrementing.

As the COVID-19 epidemic spread in 2020, many countries implemented urban lockdowns and restricted life, leading to drastic changes in the global economy and society. In 2020, the Centers for Disease Control and Prevention (CDC) FoodNet identified 26% fewer infections than the annual average from 2017 to 2019, with fewer infections linked to international travel [14]. In March 2020, due to the expansion of the epidemic, many countries adopted border closures, entry controls for non-citizens, isolation and quarantine after entry, or restrictions on residents’ activities to control the epidemic. Since March, timely border control of the global travel epidemic advisory has been upgraded to “Level 3” (travel warning). Citizens avoid all non-essential travel abroad. Those arriving from abroad are required to undergo a 14-day home quarantine. Human-to-human infectious diseases may spread through international travel and tourism, but international travel and tourism decreased after the COVID-19 outbreak. The number of non-national international tourist arrivals worldwide has dropped by 74% compared with the number in 2019. The epidemic has been better controlled in Taiwan, and even domestic countries have imposed restrictions on travel, activities, gatherings, and other measures to prevent the spread of the epidemic [27]. The restrictive protective measures, including social distancing, wearing masks, and enhanced hand hygiene, have led to changes in lifestyle, behavior, and social habits that were reduced. The diseases that occurred included COVID-19 and FBD.

Food safety is related to public health and people’s livelihoods. Protecting people from the risk of poison and disease, improving nutrition, and promoting health are among the most critical issues around the world. Therefore, the government implements interdisciplinary management of food quality, including quality inspection, monitoring, proper handling, packaging, and preservation of food in accordance with international regulations and standards, industry self-regulation, and private participation to achieve food safety [28]. Education and awareness campaigns can help communicate these important messages to the public to reduce the risk of FBD. Continued public health measures such as mask-wearing and hand hygiene are crucial in preventing the spread of viral infections. Monitoring and analyzing such trends can help guide targeted interventions to further reduce the risk of FBD. Understanding these temperature-related associations is valuable for public health planning and interventions to mitigate the risk of FBD. The strategies employed to control the COVID-19 pandemic may have inadvertently contributed to reducing the incidence of FBD.

There are some limitations to this study. First, foodborne pathogens, including viruses, bacteria, and parasites, were biological agents that could cause FBD events. An FBD outbreak is defined as two or more similar cases of illness resulting from the ingestion of common foods [29]. We use data reported by Taiwan’s Ministry of Health and Welfare, which includes the entire reported population in Taiwan. Outpatient and emergency medical treatment in Taiwan is relatively convenient, and some patients do not need to report to government departments for individual treatment. Strict hygiene and social distancing measures during the COVID-19 pandemic that induced medical searches for healthcare have declined. Because many foodborne pathogens cause mild symptoms, the patient saw a doctor in a clinic rather than a hospital, resulting in underreporting of epidemiological data [30]. It was similar to or underestimated the prevalence of FBD in countries like Brazil [31]. Second, the Chinese food was mixed, and pathogens detected were delayed for days that caused the source, and pathogens were not easily detected. There were 2853 cases (overall: 6935) in 2019 and 948 cases (overall: 4920) in 2020, with a higher proportion of unidentified categories of food source in the surveillance values. There were 3352 cases (5658 cases detected) in 2019 and 1464 cases (3127 cases detected) in 2020, with only half of the case reports in all datasets in our study coming from bacterial sources. However, it does not affect the observational value of FBD. Third, other viral or parasitic pathogens and chemical agents are not available. This suggests that the government must collect these pathological causes of FBD and conduct an in-depth analysis. 

## 5. Conclusions

This study used an open dataset and showed a reduction in the occurrence of FBD, food source classifications, and pathogenic bacteria beginning with the COVID-19 era in Taiwan. The incidence of FBS showed a decrease under the prevention policies for severe respiratory infections in Taiwan. This underscores the interconnectedness of public health measures and their potential impact on various health outcomes beyond the primary target. The higher air temperature was related to FBD occurrences, and the citizens need to store and cook food correctly. The cold air temperature is related to viral FBD; more aggressive preventive measures need to be taken. Some notifiable infectious diseases, rather than those classified as FBD, also cause foodborne problems and need to be reclassified for further study. 

## Figures and Tables

**Table 1:** The places of foodborne disease acquisition from 2019 to 2020 in Taiwan.

Places	2019 FBD Cases (6935)	2020 FBD Cases (4920)	*p*	Odds Ratio (95% C.I.)	*p*
Home	324	204	<0.001 *	0.88(0.74–1.06)	0.172
	(4.7%)	(4.1%)			
Restaurant	1593	2197		2.71(2.5- 2.93)	<0.001 *
	(23%)	(44.7%)			
School	4010	1785		0.42(0.39–0.45)	<0.001 *
	(57.8%)	(36.3%)			
Office	407	343		1.2(1.04–1.39)	0.015 *
	(5.9%)	(7%)			
Hospital	145	6		0.06(0.03–0.13)	<0.001 *
	(2.1%)	(0.1%)			
Transportation vehicles	0	0			
	(0%)	(0%)			
Military facilities	19	114		8.63(5.31–14.05)	<0.001 *
	(0.3%)	(2.3%)			
Outdoor	82	6		0.1(0.04 to 0.23)	<0.001 *
	(1.2%)	(0.1%)			
Vendors	222	45		0.28(0.22–0.39)	<0.001 *
	(3.2%)	(1%)			
Exterior	92	28		0.43(0.28–0.65)	<0.001 *
	(1.3%)	(0.6%)			
Prison	62	124		2.87(2.11–3.9)	<0.001 *
	(**0.9**%)	(2.5%)			
Social welfare organization	75	45		0.84(0.58–1.22)	0.372
	(1.1%)	(0.9%)			
Others	95	63			
	(1.4%)	(1.3%)			

* p < 0.05; FBD: foodborne disease; C.I.: confidence interval.

**Table 2:** The causes of foodborne diseases from 2019 to 2020 in Taiwan.

Bacterial Causes	Odds Ratio (95% C.I.)	*p*
Bacteria		
*Vibrio vulnificus*	1.98(1.62–2.43)	<0.001 *
*Salmonella*	2.13(1.85–2.44)	<0.001 *
Enteropathogenic *Escherichia coli*	0.02(0.01–0.09)	<0.001 *
*Staphylococcus aureus*	0.99(0.86–1.14)	0.885
*Bacillus cereus*	0.38(0.34 to 0.43)	<0.001 *
Botox		
*Clostridium perfringens*		
Virus		
Norovirus	1.05(0.97–1.13)	0.249
rotavirus	0.19(0.07–0.55)	0.002 *
Natural toxin		
Plant	8.47(2.55–28.2)	<0.001 *
Histamine	0.24(0.1–0.57)	0.002 *
Puffer poison		
Others		

* p < 0.05, FBD: foodborne disease, C.I.: confidence interval.

**Table 3:** The relative risk of air temperature and relative humidity in viral foodborne disease events, bacterial foodborne disease events, and overall foodborne disease cases.

	Crude Relative Risk		*p*	Adjusted Relative Risk		*p*
Viral FBD events						
Air temperature	0.953(0.927–0.979)		<0.001 *	0.954(0.926–0.984)		0.003 *
Relative humidity	1.029(1–1.058)		0.046 *	1.005(0.973–1.039)		0.76
Bacterial FBD events						
Air temperature	1.07(1.034–1.107)		<0.001 *	1.078(1.034–1.124)		<0.001 *
Relative humidity	0.969(0.932–1.007)		0.112	1.015(0.971–1.061)		0.516
Overall FBD cases						
Air temperature	1.026(1.022–1.03)		<0.001 *	1.055(1.05–1.061)		<0.001 *
Relative humidity	1.027(1.023–1.032)		<0.001 *	1.058(1.053–1.063)		<0.001 *

* *p* < 0.05, FBD: foodborne disease.

## Data Availability

The datasets used in the current study are available from the correspondence.

## Supplementary Materials

The following supporting information can be downloaded at: https://www.mdpi.com/article/10.3390/medicina60030505/s1, Table S1: The events of place in foodborne disease during 2019–2020 in Taiwan; Table S2: The food source of foodborne diseases from 2019 to 2020 in Taiwan; Table S3: The events about causes of foodborne diseases from 2019 to 2020 in Taiwan.
