# Supplementary material for: Impact of Foodborne Disease in Taiwan during the COVID-19 Pandemic
Source: Medicina (Kaunas). 2024 Mar 19;60(3):505. doi: 10.3390/medicina60030505 (PMC10972290; doi:10.3390/medicina60030505)
Supplement: Supplementary file 1 [file medicina-60-00505-s001.zip › medicina-2868738-supplementary.pdf]

Table S1. The events of place in foodborne disease during 2019-2020 in Taiwan.

Places	2019 FBD events (n=502)	2020 FBD events (n=506)	***p***
Home	46 (9.2%)	69 (13.6%)	0.052
Restaurant	310 (61.8%)	313 (61.9%)	
School	86 (17.1%)	68 (13.4%)	
Office	15 (3%)	19 (3.8%)	
Hospital	7 (1,4%)	1 (0.2%)	
Transportation vehicles	0 (0%)	0 (0%)	
Military facilities	3 (0.6%)	8 (1.6%)	
Outdoor	5 (1%)	2 (0.4%)	
Vendors	16 (3,2%)	7 (1.4%)	
Exterior	2 (0.4%)	2 (0.4%)	
Prison	3 (0.6%)	6 (1.2%)	
Social welfare organization	6 (1.2%)	4 (0.8%)	
Others	7 (1.4%)	7 (1.4%)	

*p<0.05; FBD: foodborne disease

Table S2. The food source of foodborne diseases from 2019 to 2020 in Taiwan

Food media	2019 FBD events (45)	2020 FBD events (37)	*p*
Seafood	11	4	0.075
Aquatic processing	2	0	
Meat and processed meat products	3	1	
Eggs and processed egg products	2	0	
Cereals and processed cereal products	0	1	
Fruits and vegetables and their processed products	5	5	
Cakes, candy	1	2	
Boxed meals	22	19	
Compounded foods	3	5	
Others	1	1	

*$p<0.05$; FBD: foodborne disease

There was 1 event involving aquatic products and compound prepared foods. There was 1 event involving processed aquatic products and boxed meals. There was 1 event involving meat and its processed products and fruits and vegetables and their processed products. There was 1 event involving boxed meals and other foods. There was 1 event involving meat and its processed products and boxed meals.

Table S3. The events about causes of foodborne diseases from 2019 to 2020 in Taiwan.

Bacterial causes	2019 FBD events (*n*=502)	2020 FBD events (*n*=506)	*p*
Bacteria	91	80	0.037*
Vibrio vulnificus	10	18	
Salmonella	21	26	
Enteropathogenic *Escherichia coli*	3	1	
Staphylococcus aureus	35	17	
Bacillus Cereus	33	22	
Botox	0	1	
Clostridium perfringens	2	0	
Virus	142	122	0.653
Norovirus	140	121	
rotavirus	2	1	
Natural toxin	12	10	0.118
Plant	2	4	
Histamine	8	4	
Puffer poison	0	2	
Others	2	0	
Unknow	290	294	

*$p<0.05$; FBD: foodborne disease.

A total of 29 events were caused by combined bacteria and viruses. There were 2 events caused by combined bacteria and natural poisons. There was 1 event caused by combined viruses and natural poisons.
